# Prevalence Rates and Characteristics of Malnutrition, Frailty, and Other Nutrition and Muscle Mass-Related Conditions Document Potential Quality of Care Gap for Medicare Patients in US Skilled Nursing Facilities

**DOI:** 10.3390/geriatrics7020042

**Published:** 2022-03-31

**Authors:** Mary Beth Arensberg, Cory Brunton, Susan Drawert, Brenda Richardson

**Affiliations:** 1 Abbott Nutrition Division of Abbott, Columbus, OH 43219, USA; cory.brunton@abbott.com; 2 Abbott, Minneapolis, MN 55402, USA; susan.drawert@abbott.com; 3 Brenda Richardson LLC, Salem, IN 47167, USA; brenda@brendarichardson.com

**Keywords:** medicare diagnosis claims, skilled nursing facility (SNF), COVID-19, malnutrition, frailty, length of stay, discharges, total charges

## Abstract

Changes to the payment structure of the United States (U.S.) healthcare system are leading to an increased acuity level of patients receiving short-term skilled nursing facility care. Most skilled nursing facility patients are older, and many have medical conditions that cannot be changed. However, conditions related to nutrition/muscle mass may be impacted if there is early identification/intervention. To help determine the diagnosis and potential impact of nutrition/muscle mass-related conditions in skilled nursing facilities, this study evaluated 2016–2020 US Medicare claims data. Methods aimed to identify a set of skilled nursing facility claims with one or more specific diagnoses (COVID-19, malnutrition, sarcopenia, frailty, obesity, diabetes, and/or pressure injury) and then to determine length of stay, discharge status, total charges, and total payments for each claim. Mean values per beneficiary were computed and between–group comparisons were performed. Results documented that each year, the total number of Medicare skilled nursing facility claims declined, whereas the percentage of claims for each study diagnosis increased significantly. For most conditions, potentially related to nutrition/muscle mass, Medicare beneficiaries had a shorter length of skilled nursing facility stays compared to those without the condition(s). Furthermore, a lower percentage of these Medicare beneficiaries were discharged home (except for those with claims for sarcopenia and obesity). Total claim charges for those with nutrition/muscle mass-related conditions exceeded those without (except for those with sarcopenia). We conclude that although the acuity level of patients in skilled nursing facilities continues to increase, skilled nursing facility Medicare claims for nutrition/muscle mass-related conditions are reported at lower levels than their likely prevalence. This represents a potential care gap and requires action to help improve patient health outcomes and skilled nursing facility quality metrics.

## 1. Introduction

The World Health Organization (WHO) has recognized that “long-term-care systems enable older people, who experience significant declines in capacity, to receive the care and support that allow them to live a life consistent with their basic rights, fundamental freedoms, and human dignity” [1]. In the United States (U.S.), long-term care is typically provided by skilled nursing facilities, and the acuity level of patients receiving short-term skilled nursing care has risen in the last decade, thus, the range of services provided by skilled nursing facilities has expanded [2]. Such trends in the US are driven in part by the country’s evolving healthcare payment structure and a focus on decreasing the length of hospital stays, leading to more patients being discharged to post-acute care to avoid prolonged hospitalizations. 

The majority of skilled nursing facility patients are older adults [3], and many older Americans have multiple chronic diseases, disabilities, and other conditions [4] that can increase their risk for both higher acuity levels and poorer health outcomes, regardless of care setting. Although a number of these medical conditions are not modifiable, those related to nutrition and muscle mass can often be changed if there is early identification and intervention [5]. Furthermore, as identified by the WHO, there are strong associations between conditions such as malnutrition (specifically undernutrition) and premature mortality, poor quality of life, and reduced functional ability [6]. However, across the spectrum of care, conditions such as malnutrition, sarcopenia, and frailty are frequently not diagnosed [7], which can represent a potential gap in care.

To help better define how this gap may exist in skilled nursing facilities—both in terms of whether nutrition/muscle mass-related conditions are diagnosed and their potential impact—we evaluated recent US Centers for Medicare & Medicaid Services Medicare claims data. Medicare is the US federal government healthcare program for older adults and individuals with disabilities and certain conditions. This article reviews 2020 Medicare skilled nursing facility claims for COVID-19, and 2016–2020 claims for conditions potentially related to nutrition and/or muscle mass (malnutrition, sarcopenia, frailty, obesity, diabetes, pressure injury). Specific characteristics of the Medicare claims for beneficiaries with these defined conditions are also included.

## 2. Methods

### 2.1. Data Source

Data were obtained from the publicly available skilled nursing facility Centers for Medicare & Medicaid Services Standard Analytic Files [8], using information for the calendar years 2016–2020 and representing a total of 23,395,438 Medicare claims for skilled nursing facility stays. US Medicare covers health care services for individuals aged 65 and over, those with certain disabilities such as blindness, and individuals with end stage renal disease. The claims in the Standard Analytic Files were submitted by skilled nursing facilities to bill Medicare for care and services delivered during each beneficiary’s individual skilled nursing facility stay. Claim forms represent all beneficiary information recorded in skilled nursing facilities’ medical/health records that are required for billing purposes. This includes demographic information about each beneficiary, as well as other standardized information about the beneficiary’s diagnosed medical conditions, specific therapy requirements, and care needs during skilled nursing facility stays.

### 2.2. Study Population

The study population was fee-for-service Medicare beneficiaries who had one or more skilled nursing facility claim for a diagnosis of interest submitted to the Centers for Medicare and Medicaid Services claims system for the calendar years 2016–2020 (n = 9,365,419). The claims for diagnoses of interest were identified using International Classification of Diseases (ICD) 10th Edition diagnosis codes (Appendix A Table A1). The analysis is limited to the diagnosis (ICD 10 code) information submitted as a claim to the Centers for Medicare & Medicaid Services; therefore, it is unknown how the conditions studied were actually diagnosed, or how the specific diagnostic criteria were used at individual skilled nursing facilities.

### 2.3. Study Design and Analysis

Using the total skilled nursing facility Medicare claims data submitted to the Centers for Medicare & Medicaid Services in calendar years 2016–2020, a defined set of all skilled nursing facility claims with one or more of the following diagnoses was identified:

- COVID-19;
- malnutrition;
- sarcopenia;
- frailty;
- obesity;
- diabetes;
- and/or pressure injury.

For each claim, length of stay, discharge status, total charges, and total payments were determined.

### 2.4. Statistics

Medicare claims were totaled when necessary to analyze episodes of care by individual beneficiaries, then mean values per beneficiary were computed for each variable. Between–group comparisons were performed by independent t-tests on continuous variables, and chi-square tests were performed on categorical variables using SAS 9.4 software (SAS Institute, Inc, Cary, NC, USA); *p* values ≤ 0.05 were considered statistically significant.

## 3. Results

During the 2016–2020 timeframe, a defined set of 9,365,419 Medicare skilled nursing facility claims were identified that met the study design criteria of one or more specific diagnoses (COVID-19, malnutrition, sarcopenia, frailty, obesity, diabetes, and/or pressure injury). The average age of patients whose data were included in this defined set of Medicare skilled nursing facility claims was 76.8 years; 59.7% were female and 79.9% were white (Table 1). 

Over time, the total number of Medicare skilled nursing facility claims declined each year, from 5,135,377 total claims in 2016 to 4,104,986 total claims in 2020 (Table 2). Conversely, during this same time period, the percentage of claims for each study diagnosis increased significantly each year, with the percentage of malnutrition diagnosis claims more than doubling from 2019 to 2020. In 2020, of the skilled nursing facility claims for each study diagnosis, diabetes was the most frequent (34.1% of claims), followed by COVID-19 (15.3% of claims), malnutrition (11.9% of claims), obesity (10.7% of claims), and pressure injury (5.7% of claims). Claims for the other two specific study diagnoses, frailty and sarcopenia, remained low (<2.0% of claims) (Table 2).

Between 2016–2020, Medicare beneficiaries with claims for most of the individual conditions potentially related to nutrition/muscle mass (malnutrition, sarcopenia, frailty, obesity, diabetes, and/or pressure injuries) had a shorter length of skilled nursing facility stays compared to those without claims for the condition(s). Frailty and pressure injury were the exception (without claim for frailty, 41.1 days vs. with claim for frailty, 45.9 days, *p* value < 0.001; without claim for pressure injury, 41.0 days vs. with claim for pressure injury, 45.4 days, *p* value < 0.001). However, a lower percentage of beneficiaries with claims for the specific study diagnoses were discharged home except for those with claims for sarcopenia and obesity (36.7% vs. 38.3%; 48.1% vs. 48.5%, *p* value < 0.001, respectively). From 2016–2020, the percentage of all beneficiaries with claims for a nutrition/muscle mass-related condition who were discharged home decreased significantly (2016: 39.6%; 2020: 28.4%, *p* value < 0.001) (Appendix A Table A2). Furthermore, significantly more beneficiaries with claims for COVID-19 and/or nutrition/muscle mass-related conditions were discharged to short-term hospitals, compared to those without claims, except for those with claims for sarcopenia (Table 3). Total claim charges for those with claims for specific nutrition/muscle mass-related conditions exceeded those without, except for those with claims for sarcopenia (Table 3). All beneficiaries with claims for nutrition/muscle mass-related conditions had a higher mortality when compared to those without, except for those with claims for obesity or diabetes (Table 3). The percentage of deceased beneficiaries discharged from skilled nursing facilities consistently decreased from 2016–2019, but increased significantly in 2020 (Appendix A Table A2).

## 4. Discussion

Long-term care for older adults around the world can range from traditional health services provided in an institutional setting to community-based assistive care services such as caregiving and social support [1]. In the US, skilled nursing facility care involves direct care by or under the supervision of skilled nursing and therapy professionals and is provided by nursing homes and other care facilities. Although skilled nursing facility care can include both long-term residential care and short-term post-acute or rehabilitative care, the US Medicare program just covers skilled nursing facility care on a short-term basis (up to 100 days in a benefit period) following a hospitalization [9]. About one in five Medicare beneficiaries are admitted to a skilled nursing facility following a hospital stay [10].

### 4.1. Skilled Nursing Facility Medicare Claims and Beneficiary Demographics

US Medicare skilled nursing facility claims have declined in the last decade [11], and the current study also documented year-over-year decreases in skilled nursing facility Medicare claims for a set of specific diagnoses. In addition, the demographics of the current study’s Medicare claim set of specific diagnoses were generally comparable to those reported for the skilled nursing facility patient population as a whole. According to US Centers for Disease Control and Prevention data, the majority of patients in US nursing homes are female (64.6%), white (75.1%), and older [3]; the current study’s five-year Medicare claim set included a slightly lower female (60.35%), and higher white (82.74%), percentage of patients. 

### 4.2. Skilled Nursing Facility Patient Acuity and Prevalence Rates of Specific Conditions

Globally, groups such as the World Economic Forum Global Coalition for Value in Healthcare are increasing their efforts to accelerate development of value-based health systems and pilot innovative new approaches to person-centered care [12]. In 2019, the US Centers for Medicare & Medicaid Services moved to a new Payment Driven Payment Model for skilled nursing facility care. This new payment model focuses on quality of care and patient outcomes rather than the quantity of provided services which was the basis for the Centers for Medicare & Medicaid Services’ previous skilled nursing facility care payment model. The new Payment Driven Payment Model incentivizes skilled nursing facility providers to specialize in providing care for more complex clinical conditions [10]. Indeed, skilled nursing facilities are increasingly caring for patients with a higher level of nursing and rehabilitation needs [2].

Similarly, the current study’s results documented that over time, there was an increasingly higher percentage of Medicare claims for the specific diagnoses we investigated, potentially reflecting higher skilled nursing facility patient acuity. The percentage of patients being discharged home from skilled nursing facilities was decreasing, whereas the mortality rate and percentage of patients with COVID-19 and nutrition/muscle mass-related conditions being discharged to short-term hospitals was increasing; therefore, this could be another indicator of increased acuity. A further point to consider is that under the US Payment Driven Payment Model, the diagnoses coded on the five-day/Initial Minimum Data Set (completed when patients are first admitted to skilled nursing facilities) are factored into the payment for a patient’s entire skilled nursing facility stay. Thus, accurate recording of diagnoses and supporting documentation have been identified as paramount to ensure appropriate payment [13]. This need to quickly and accurately identify diagnoses may have partially accounted for the increases we observed in 2019, and particularly 2020 (the new Payment Driven Payment Model became effective beginning October 1, 2019 [14]), for the specific Medicare claims included in our study.

In the US, COVID-19 was reported for the first time in a 2020 skilled nursing facility Centers for Medicare & Medicaid Services claims data set, which identified 762,594 beneficiaries who were confirmed to have COVID-19, and an additional 532,901 who were likely to have it, meaning COVID-19 was suspected but not confirmed by a positive test result [15]. These data included beneficiaries who were diagnosed with COVID-19, or likely COVID-19, in a skilled nursing facility, as well as those diagnosed in the hospital or other care settings after being transferred from the skilled nursing facility. Our analysis found 626,956 skilled nursing facility claims in 2020 with a specific COVID-19 ICD-10 diagnosis listed on the claim. We were not able to include any additional parameters such as diagnosis confirmation or COVID-19 cases that may have started in the skilled nursing facility but were not diagnosed until hospitalization. It is likely that these, as well as other logistical challenges in COVID diagnosis procedures, especially the fact that Centers for Medicare & Medicaid guidance on COVID-19 diagnosis coding was not available to skilled nursing facilities until March 2020 [16], may explain the difference in case numbers. It is also likely that COVID-19 impacted the increase in deaths and decreased the number of discharges home we observed from 2019 to 2020.

Nutrition and muscle mass-related conditions are global concerns. The United Nations Sustainable Development Goal Target 2.2 aims to end all forms of malnutrition and address the nutrition needs of specific populations by 2030, including older adults [17]. In addition, the WHO recommends “oral supplemental nutrition for those affected by undernutrition” [18]. The WHO developed its Integrated Care for Older People initiative [19] to address conditions impacting intrinsic capacity, including frailty and sarcopenia [20], and also has programs focused on obesity [21], diabetes [22], and pressure injuries [23]. In the US, post-acute care settings for nutrition/muscle mass-related conditions can impact readmissions and complications, including pressure ulcer incidence [24], but these conditions are frequently not diagnosed [7].

We found few reported studies that specifically researched US skilled nursing facility Medicare claims for nutrition/muscle mass-related conditions. Of those identified, most considered the impact of specific conditions on health outcomes and included care settings beyond skilled nursing facilities. Cawthon et al. investigated sarcopenia and health care utilization (including skilled nursing care) in older women, and in general, they did not find evidence of increased healthcare utilization for those classified with sarcopenia [25]. Similarly, in our study, we found that Medicare beneficiary claims for sarcopenia were not different from those without the diagnosis in terms of patients being discharged home or to short-term hospitals, or for total claim charges, whereas for other nutrition/muscle mass-related conditions there were differences. Kosar et al. investigated the effect of obesity on post-acute outcomes of skilled nursing facility patients with hip fractures, and concluded that obesity was associated with worse outcomes, including increased hospital readmission [26]. In our study, those with a diagnosis of obesity, compared to those without the condition, had higher rates of discharge to short term hospitals and higher total claim charges.

When considering how our research may compare to other studies identifying nutrition/muscle mass-related conditions in skilled nursing facility populations, the research literature typically reflects much higher prevalence rates (both in the US and globally) [27,28,29,30,31,32,33,34,35,36] than we found (Appendix A Table A3), but such research literature is not based on claims data, which is an important distinction. Medicare data—similar to other healthcare claims data—are not collected for research purposes but rather to document the provision of healthcare services for billing purposes, which can affect estimates of both disease incidence and prevalence [37].

### 4.3. Skilled Nursing Facility Quality of Care Measures and Health Outcomes

Quality of care in long-term care and skilled nursing continues to be an important area of focus both globally [1] and in the US [38]. The US Centers for Medicare & Medicaid Services have created the skilled nursing facility Quality Reporting Program, as mandated by the Improving Medicare Post-Acute Care Transformation Act of 2014. This 2014 legislation requires skilled nursing facilities to report patient assessment data for quality measures, resource use, and other domains. Starting in the 2018 fiscal year, skilled nursing facilities that failed to submit the required quality data became subject to a two-percentage point reduction in their Annual Payment Update for all Medicare claims for that year. The Improving Medicare Post-Acute Transformation Act of 2014 also requires that the assessment data is standardized and interoperable. This allows data to be exchanged among post-acute providers and other providers, which potentially improves Medicare beneficiary outcomes through shared decision-making, care coordination, and enhanced discharge planning [39].

The skilled nursing facility US Quality Reporting Program includes required quality measures as well as data elements that must be collected and reported on for each Medicare patient stay. Skilled nursing facility performance on these measures is publicly available on the US Centers for Medicare & Medicaid Services Care Compare website [40]. Multiple quality measures, including those related to change in skin integrity, pressure injury, falls with major injury, and hospital readmission can be impacted by nutrition related conditions. For example, malnutrition is linked to an increased incidence of healthcare-acquired pressure injuries, immune suppression, increased infections, higher hospital readmission rates, and higher treatment costs [24], and similarly, sarcopenia and frailty are also associated with poorer health outcomes [5]. However, only the quality measure for long-stay patients with high-risk/unstageable pressure ulcers specifically includes malnutrition as a criterion for defining high risk [41], thus representing a potential opportunity for policy development. There is evidence, however, of potential change, as nutritional status and frailty were both mentioned in the 2022 skilled nursing facility Quality Reporting Program’s final rule, as conditions that the US Centers for Medicare & Medicaid Services may consider in future measure development efforts [42].

Under the US Payment Driven Payment Model there is greater focus on skilled nursing facility health outcomes. For example, Medicare can withhold up to 2% of reimbursements for skilled nursing facilities with high hospital readmission rates. In the 2019 fiscal year, 20% of skilled nursing facilities had the maximum amount of reimbursement withheld [43]. For all the specific diagnoses we considered except sarcopenia, there was a higher percentage of Medicare claims where beneficiaries with the condition were discharged to short-term hospitals, and a lower percentage were discharged home. This has implications for patient health outcomes and reimbursement, and reinforces the need to identify, intervene in, and diagnose nutrition/muscle mass-related conditions. When these conditions are identified, they must be documented as a diagnosis within five days of patient admission to the skilled nursing facility, as treatment delays result in worse outcomes and increased costs can occur. Furthermore, if these conditions are not documented, there may be a direct impact on the resources needed to effectively address patient nutrition requirements.

Except for diabetes, all of the other nutrition/muscle mass-related conditions we investigated had much lower rates of diagnosis on skilled nursing facility Medicare claims than their possible prevalence rates in this care setting, representing a potential care gap. This could occur for several reasons. First is increased patient acuity. Only a limited number of diagnoses (specifically 8–12 diagnoses [44]) can be submitted on US Centers for Medicare & Medicaid Services claims. For patients with higher acuity levels, other conditions may have been prioritized for claims reporting over the specific diagnoses we considered. A second reason that possibly contributes to a potential care gap is staffing. Unlike acute care where registered dietitian nutritionists are available full-time to provide nutritional care, most skilled nursing facilities only have a registered dietitian nutritionist onsite for a specific number of hours per month. This means the registered dietitian nutritionist must rely on the interdisciplinary team to identify patients at risk for nutrition/muscle mass-related conditions. In addition, the registered dietitian nutritionist must communicate effectively with the medical director to ensure that these conditions are accurately diagnosed and have appropriate medical record documentation [45]. Another reason for the potential care gap could be lack of awareness. Conditions such as malnutrition are a significant comorbidity, affecting survival and healthcare costs. However, even in the hospital setting, fewer than 9% of patients have a malnutrition diagnosis at discharge [46], whereas an estimated 20–50% of patients are malnourished on admission [47].

The need to better identify, intervene in, and document diagnoses for nutrition/muscle mass-related conditions also points to a potential opportunity for implementing nutrition-focused quality improvement programs to help improve outcomes and control costs. Quality improvement programs can help improve care processes to benefit both patients and health systems. For example, a recently published nutrition-focused quality improvement program in the outpatient setting in Colombia was associated with improvements in nutritional outcomes and muscle mass [48].

Nutrition-focused quality improvement programs for malnutrition typically include: (1) systematically screening all patients for malnutrition or risk for malnutrition, (2) documenting malnutrition diagnoses, (3) intervention with oral nutrition supplements in addition to exercise and nutrition education or counseling, (4) and continuous follow-up [49]. Reportedly, there are very few published nutrition-focused quality improvement programs in skilled nursing facilities [45]. In other US healthcare settings, nutrition-focused quality improvement programs have been associated with overall cost-savings of over $4.8 million in the hospital setting [50], and a cost-savings of $2.3 million in the home health setting [51], which is driven by reduced healthcare resource use (combination of hospitalizations, emergency department visits, and outpatient visits) over a study period of 90 days.

### 4.4. Strengths and Limitations

This is one of the first studies, to our knowledge, that reports US Centers for Medicare & Medicaid Services claims data for COVID-19 and specific nutrition and muscle mass-related diagnoses. The results demonstrate that claims rates are lower than potential prevalence rates, which points to a potential care gap in the diagnosis of nutrition and muscle mass-related conditions. One of the limitations of our research is that it is a descriptive study, and the basis for the US Centers for Medicare & Medicaid Services claims and their outcome characteristics could not be identified. Another limitation is that implementation of the US Payment Driven Payment Model in 2019, followed quickly by the COVID-19 pandemic, presented many challenges to skilled nursing facilities that may have impacted who was admitted, the specific care and services provided, coding processes, and a host of other operational procedures, making it difficult to draw firm conclusions [16]. However, these limitations provide opportunities for further investigation of any new trends and validation of our study findings.

## 5. Conclusions

As the acuity level of patients in US skilled nursing facilities continues to increase, nutrition and muscle mass-related conditions are not being reported on US Centers for Medicare & Medicaid Services skilled nursing facilities claims at levels that reflect the likely prevalence of these conditions. This represents a potential care gap that merits attention, since these conditions alone or combined can negatively impact patient health outcomes, as well as facility performance on associated quality metrics. The US Centers for Medicare & Medicaid Services, similarly to other healthcare systems around the globe, is continuing to move toward a value-based care approach focused on quality care. Thus, accurately documenting, and addressing nutrition and muscle mass-related conditions more quickly, is critical to achieving overall goals for improving patient and quality outcomes and reducing costs.

## Figures and Tables

**Table 1:** Demographic characteristics for the skilled nursing facility US Medicare claim set of specific diagnoses * (n = 9,365,419).

Characteristic	Percent by Diagnosis							
	All Claims (n = 9,365,419)	COVID-19 (n = 626,956)	Malnutrition (n = 1,164,000)	Sarcopenia (n = 24,900)	Frailty (n = 344,721)	Obesity (n = 1,427,650)	Diabetes (n = 6,862,066)	Pressure Injury (n = 1,060,660)
Female	59.7%	61.7%	56.7%	60.4%	63.0%	65.7%	55.7%	54.4%
White	79.9%	78.4%	79.4%	82.4%	83.9%	83.0%	77.0%	74.9%
Age (years)	76.8	77.9	77.1	78.5	79.9	72.2	75.8	76.5

* Based on total Medicare fee-for-service claims for specific diagnoses submitted to the US Centers for Medicare & Medicaid Services for care provided during the calendar years 2016–2020.

**Table 2:** Percentage of diagnosed conditions by year for the skilled nursing facility US Medicare claim set of specific diagnoses * (n = 23,395,438).

Year	Total Number of Claims	Percent by Diagnosis						
		COVID-19	Malnutrition	Sarcopenia	Frailty	Obesity	Diabetes	Pressure Injury
2016	5,135,377	-	2.6%	0.0%	1.3%	4.2%	27.3%	4.0%
2017	4,984,214	-	3.0%	0.0%	1.4%	4.5%	27.7%	4.1%
2018	4,738,799	-	3.3%	0.0%	1.5%	4.9%	28.2%	4.2%
2019	4,432,062	-	5.3%	0.2%	1.5%	7.1%	30.2%	4.8%
2020	4,104,986	15.3%	11.9%	0.4%	1.7%	10.7%	34.1%	5.7%

* Based on total Medicare fee-for-service claims for specific diagnoses submitted to the US Centers for Medicare & Medicaid Services for care provided during the calendar years 2016–2020; all *p* values < 0.0001.

**Table 3:** Outcome characteristics for skilled nursing facility US Medicare claims for a set of specific diagnoses * (n = 9,365,419).

Characteristic	Diagnosis														
	All	COVID-19 ^†^		Malnutrition		Sarcopenia		Frailty		Obesity		Diabetes		Pressure Injury	
		No	Yes	No	Yes	No	Yes	No	Yes	No	Yes	No	Yes	No	Yes
Mean length of stay in days	41.8	40.9	50.2	41.4	38.0	41.2	38.4	41.1	45.9	41.5	37.9	41.9	39.7	41.0	45.4
Mean total payment	$14,812	$14,016	$17,046	$14,007	$15,941	$14,109	$14,510	$14,101	$14,647	$14,063	$14,782	$13,971	$14,426	$13,931	$17,812
Mean total charges	$22,476	$21,884	$21,400	$21,786	$23,355	$21,869	$21,693	$21,853	$22,946	$21,773	$23,243	$21,603	$22,476	$21,613	$27,167
Percent discharged home	34.8%	37.5%	10.6%	37.2%	27.3%	36.7%	38.3%	36.7%	33.5%	48.1%	48.5%	37.5%	34.8%	37.4%	22.5%
Percent discharged short-term hospital	22.9%	22.5%	16.5%	22.1%	25.7%	22.3%	21.0%	22.3%	22.7%	22.3%	22.5%	21.0%	25.4%	21.9%	32.2%
Percent discharged dead	3.8%	3.1%	6.9%	3.1%	5.4%	3.2%	3.6%	3.2%	3.9%	3.3%	1.8%	3.4%	2.9%	3.1%	5.8%

* Based on total Medicare fee-for-service claims provided during the calendar years 2016–2020 for specific diagnoses submitted to the US Centers for Medicare & Medicaid Services for care; *p* values < 0.001. ^†^ Only 2020 data available.

## Data Availability

See Centers for Medicare & Medicaid Services Standard Analytical Files-LDS, 2021; Available online: https://www.cms.gov/Research-Statistics-Data-and-Systems/Files-for-Order/LimitedDataSets/StandardAnalyticalFiles (accessed on 4 June 2021).

## Appendix A

**Table A1:** International Classification of Diseases (ICD) (10th Edition) diagnosis codes used to identify disease states for a skilled nursing facility US Medicare claim set of specific diagnoses *.

Diagnosis	ICD-10 Codes (Includes Any Code That Starts with the Below Characters)
COVID-19	“U071”
Malnutrition	“E40”,”E41”,”E42”,”E43”,”E44”,”E45”,”E46”
Sarcopenia	“M6284”
Frailty	“R54”
Obesity	“E65”,”E66”
Diabetes	“E08”,”E09”,”E10”,”E11”,”E12”,”E13”
Diabetic ulcer	“E11621”,”E11622”
Pressure ulcer	“L89”

* Based on total Medicare fee-for-service claims for specific diagnoses submitted to the US Centers for Medicare & Medicaid Services, for care provided during the calendar years 2016–2020.

**Table A2:** Patients discharged home or deceased for a skilled nursing facility US Medicare claim set of specific diagnoses * (n = 9,365,419).

Year	Discharged Home	Discharged Deceased
2016	39.6%	3.3%
2017	38.6%	3.1%
2018	37.9%	2.9%
2019	38.0%	2.7%
2020	28.4%	4.1%

*p* value < 0.001. * Based on total Medicare fee-for-service claims for specific diagnoses submitted to the US Centers for Medicare & Medicaid Services for care provided during the calendar years 2016–2020.

**Table A3:** Percentage of nutrition/muscle mass-related diagnoses for a skilled nursing facility US Medicare claim set of specific diagnoses * and estimated nursing home prevalence rates of the same diagnoses.

Diagnosis	Percentage Range of Diagnosis over Five-Year Period in Medicare Claim Set of Specific Diagnoses	Prevalence of Conditions in US Nursing Homes as Reported in the Research Literature	Prevalence of Conditions in Nursing Homes Globally as Reported in the Research Literature
Malnutrition	2.6–11.9%	20% [27]	Up to 50% depending on definition and cut-off values used [28]
Sarcopenia	0.0–0.4%	No prevalence estimates found	41% [29]
Frailty	1.3–1.7%	No prevalence estimates found	52.3% [30]
Obesity	4.2–10.7%	26% [31]	18.5% [32]
Diabetes	27.3–34.1%	25–34% [33]	26% [34]
Pressure injury	4.0–5.7%	2–28% [35]	3.4–32.4% [36]

* Based on total Medicare fee-for-service claims for specific diagnoses submitted to the US Centers for Medicare & Medicaid Services for care provided during the calendar years 2016–2020.
